# The prognostic value of serum CA 19-9 for patients with advanced lung adenocarcinoma

**DOI:** 10.1186/s12885-016-2897-6

**Published:** 2016-11-14

**Authors:** Yuki Sato, Daichi Fujimoto, Keiichiro Uehara, Ryoko Shimizu, Jiro Ito, Mariko Kogo, Shunsuke Teraoka, Ryoji Kato, Kazuma Nagata, Atsushi Nakagawa, Kojiro Otsuka, Hiroshi Hamakawa, Yutaka Takahashi, Yukihiro Imai, Keisuke Tomii

**Affiliations:** 1 Department of Respiratory Medicine, Kobe City Medical Center, General Hospital, 2-1-1 Minatojima-minamimachi, Chuo-ku, Kobe, 650-0047 Japan; 2 Department of Pathology, Kobe City Medical Center, General Hospital, 2-1-1 Minatojima-minamimachi, Chuo-ku, Kobe, 650-0047 Japan; 3 Department of Thoracic Surgery, Kobe City Medical Center, General Hospital, 2-1-1 Minatojima-minamimachi, Chuo-ku, Kobe, 650-0047 Japan

**Keywords:** CA 19-9, CYFRA 21-1, Lung adenocarcinoma, Tumor marker, Prognostic marker

## Abstract

**Background:**

This study aimed to assess the prognostic accuracy of serum CA 19-9 in patients with advanced lung adenocarcinoma.

**Methods:**

We retrospectively reviewed data of 246 patients who were diagnosed at our institute with advanced (stage IIIB or IV) lung adenocarcinoma between March 2006 and December 2012. We excluded patients who received no chemotherapy, or for whom we had no data on pre-treatment tumor markers. We also evaluated 116 consecutive resected specimens from patients with clinical stage I lung adenocarcinoma pathologically.

**Results:**

The 76 (31 %) patients who were CA 19-9^+^ had shorter overall survival (OS) than CA 19-9^−^ group (12.5 vs 26.2 months, *P* = 0.005). Cox’s multivariate regression analysis identified Eastern Cooperative Oncology Group Performance Status 0 or 1 (*P* < 0.001), mutated epidermal growth factor receptor (*EGFR*) status (*P* < 0.001), stage IIIB (*P* < 0.001), CYFRA 21-1^−^ (*P* < 0.001), CA 19-9^−^ (*P* = 0.005) and use of platinum doublet therapy (*P* = 0.034) as independent predictors of longer OS. We stratified patients by CA 19-9 and CYFRA 21-1 as double positive (CA 19-9^+^/CYFRA 21-1^+^, *n* = 59), single positive (either CA19-9^+^ or CYFRA 21-1^+^, *n* = 113), or double negative (CA 19-9^−^/CYFRA 21-1^−^, *n* = 74). Their respective OS were 10.0, 23.3 and 31.8 months (*P* < 0.001). Pathological analysis also correlated CA 19-9 expression with malignant features such as vessel invasion, pleural invasion, cancer invasive factors and mucin production.

**Conclusions:**

CA 19-9 and CYFRA 21-1 are independent prognostic markers in patients with advanced lung adenocarcinoma. Combined use of CA 19-9 and CYFRA 21-1 provides further prognostic information in patients with advanced lung adenocarcinoma.

**Electronic supplementary material:**

The online version of this article (doi:10.1186/s12885-016-2897-6) contains supplementary material, which is available to authorized users.

## Background

Lung cancer is the leading cause of cancer death worldwide. Unfortunately, most lung cancers are already unresectable and metastatic at initial diagnosis [1, 2]. Overall survival (OS) of patients with advanced lung adenocarcinoma (ALAD) is still very poor, despite progress in treatment and chemotherapy. ALAD prognosis can be assessed through various factors, such as pathologic characteristics, imaging features, and oncogenes, but identification of more accurate prognostic markers is imperative.

Measurement of tumor markers is a non-invasive means to predict prognosis, and is therefore used in daily clinical practice [3]. Earlier investigations of the relationships between prognosis and serum cytokeratin 19 fragments (CYFRA 21-1), carcinoembryonic antigen (CEA) or neuron-specific enolase (NSE) in ALAD patients found only CYFRA 21-1 to be an independent prognostic marker among them [4–6]. Therefore, identification of another independent prognostic tumor marker would have great value in managing these patients.

Carbohydrate antigen 19-9 (CA 19-9) is a tumor-associated antigen originally isolated from a human colorectal cancer cell line in 1979 by Koprowski [7]. Since development of radioimmunometric assays, CA 19-9 has been used to monitor various cancer types, and is used as a prognostic marker in pancreatic, colon, and stomach adenocarcinoma [8–15].

Although patients with ALAD who show extremely high serum levels of CA 19-9 are reportedly have poor prognoses, the relationship between serum CA 19-9 and prognosis in lung adenocarcinoma has not been studied yet [16, 17]. We hypothesized that CA 19-9 is a prognostic marker for ALAD patients.

The purpose of this study was to investigate the clinical utility of CA 19-9 as a prognostic marker in ALAD patients, and to improve its prognostic accuracy.

## Methods

### Study subjects

We retrospectively analyzed 433 patients diagnosed advanced (stage IIIB or IV) lung adenocarcinoma at Kobe City Medical Center General Hospital between March 2006 and December 2012. We excluded patients who received no chemotherapy (*n* = 71), or for whom no data on tumor markers (CEA, CYFRA 21-1 and CA 19-9) before receiving chemotherapy was available (*n* = 116). Patients who reported never having smoked were defined as never-smokers, those who had smoked within 1 year of the diagnosis were categorized as current smokers, and the rest were considered to be former smokers. All patients were classified by clinical stage according to the 7^th^ edition TNM (tumor, node, metastasis) classification [18]. OS was measured from the diagnosis of lung cancer until death from any cause or the end of the follow-up. We isolated tumor DNA from various specimens and analyzed epidermal growth factor receptor gene (*EGFR*) mutation status at exons 18–21, using the peptide nucleic acid-locked nucleic acid PCR clamp methods, as described previously [19]. We retrospectively analyzed the presence of intestinal pneumonia, non-tuberculous mycobacteriosis (NTM) infection, bronchiectasis, and diffuse panbronchiolitis by reviewing patients’ charts and radiological records.

### Determination of tumor markers concentration

Serum samples were obtained to determine tumor markers CEA, CYFRA 21-1 and CA 19-9 as a part of routine evaluations within 28 days before starting chemotherapy. The concentration of each tumor makers was measured using LumiPulse Presto kit (Fujirebio Inc., Tokyo, Japan). It utilizes the chemiluminescent enzyme immunoassay (CLEIA) principle and is a fully automated assay [20]. The CLEIA method uses 1.6 ng/mL as the upper limit of normal (ULN) serum CYFRA 21-1 level in healthy individuals [21]. For this study, we set the cutoff value for CYFRA 21-1 at 2.2 ng/mL, which was used in a previous study that showed the prognostic impact of CYFRA 21-1 in patients with ALAD, and is the mean value for healthy subjects +3 SD (standard deviation) [4]. The cutoff values for serum CEA and CA 19-9 were set at 5.0 ng/mL and 37.0 U/mL, which are their respective ULNs [22, 23]. This testing was performed at the Department of Laboratory Medicine at our hospital.

### Pathological analysis

**Fig. 1:**
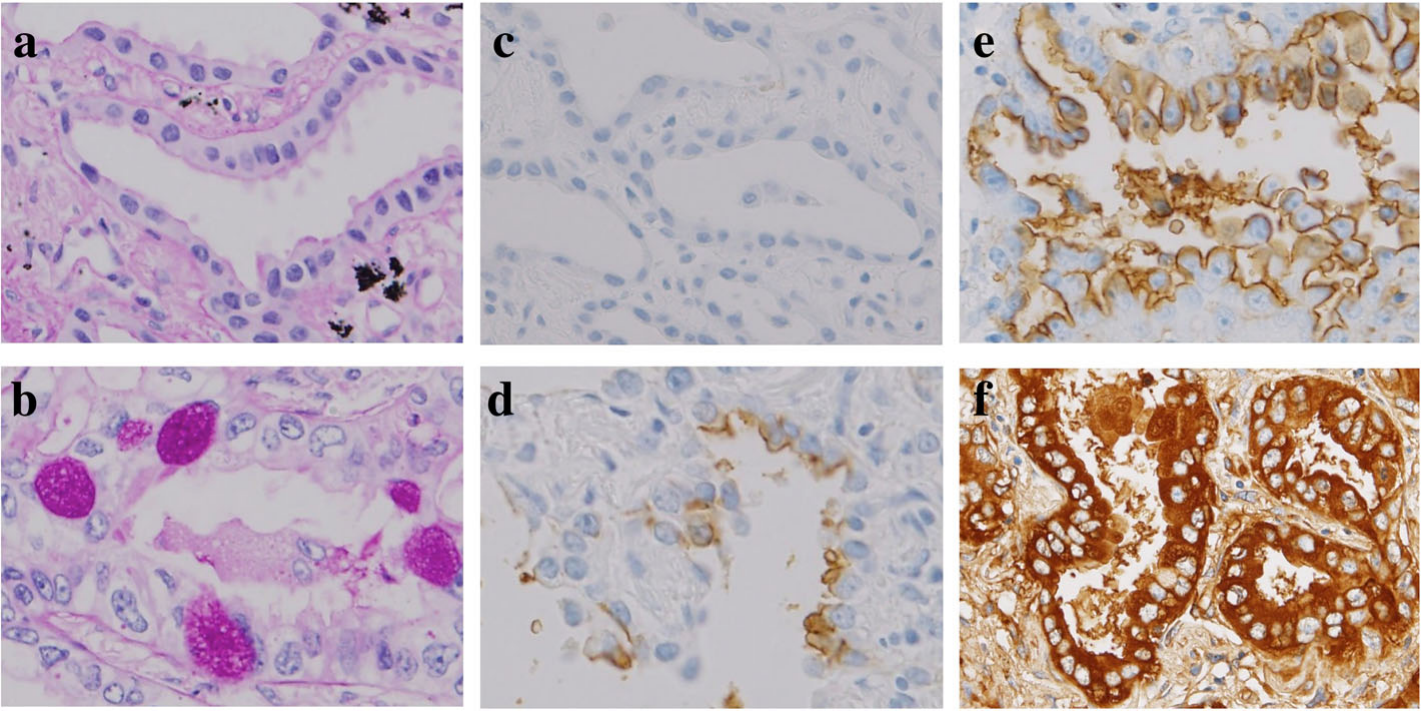
**a**, **b** Stage I adenocarcinoma specimens (diastase-resistant periodic acid Schiff stain; × 400, A: negative; B: positive). **c**–**f** Immunohistochemical staining of stage I adenocarcinoma specimens with antibodies specific for 116-NS-19-9. Representative staining patterns for **c**: intensity 0; **d**: intensity 1; **e**: intensity 2; and **f**: intensity 3 (×400)

Additionally, we retrospectively analyzed post-operative specimens from patients with clinical stage I lung adenocarcinoma who underwent surgery at our hospital between January 2008 and May 2010. We evaluated the presence of vessel invasion, pleural invasion, lymph node metastasis and postoperative pathological stage. The presence of mucin was also assessed using diastase-resistant periodic acid Schiff (D-PAS) staining in all samples with 5 % increments (Fig. 1a, b) [24, 25]. We tested CA 19-9 expression, immunohistochemically (IHC), using the 116-NS-19-9 antibody (Covance Inc., Princeton, USA). We applied the expression score, which was described previously [26–28]. Percentages of CA 19-9^+^ tumor cells (proportion score) was scored as 0: none (0 %); 1: 1–10 %; 2: 11–30 %; 3: 31–50 %; 4: 51–70 %; and 5: 71–100 % of each tumor sample. The intensity of staining (intensity score) was scored as 0: no staining; 1: weak staining; 2: moderate staining; and 3: strong staining in >10 % of cancer cells (Fig. 1c–f). The proportion score and intensity score were added to yield a total expression score of 0–8; samples that scored ≥ 3 were defined as CA 19-9^+^. We defined cases with at least one of the pathologic invasive factors—vessel invasion, pleural invasion, or lymph node metastasis—as positive for cancer invasive factors [29]. All pathological analyses were evaluated by two experienced pathologists who were unaware of the patients’ conditions.

### Statistical analysis

Continuous variables were analyzed using Student’s t-test. Dichotomous variables were analyzed using Fisher’s exact test. Correlations between CA 19-9 levels and CYFRA 21-1 levels were assessed using Spearman’s rank-based correlation test. The Kaplan − Meier method was used to estimate survival outcomes; groups were compared using the log-rank test. Cox’s proportional hazard models were fitted to determine associations between patient characteristics and survival outcomes. A multivariate Cox proportional hazard model was developed on all clinically important factors (age, sex, smoking status, ECOG PS, *EGFR* status, stage, positivity of serum CEA, CYFRA 21-1 and CA 19-9, and platinum doublet therapy administration). The results are expressed as hazard ratios (HRs) with 95 % confidence intervals (CI). All tests were two-tailed. A value of *P* < 0.05 was considered to indicate significance. We conducted statistical analyses on JMP software (11^th^ version; SAS Institute, Cary, NC, USA).

## Results

### Patient characteristics

**Fig. 2:**
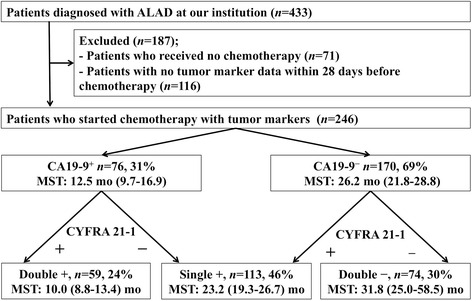
Patient selection and exclusion criteria. Patients were stratified into 3 groups by their serum tumor markers: double positive (Double +): CA 19-9^+^/CYFRA 21-1^+^; single positive (Single +): either CA 19-9^+^ or CYFRA 21-1^+^; and double negative (Double −): CA 19-9^−^/CYFRA 21-1^−^. Median survival time of each group is indicated in months (with ranges). ALAD: advanced lung adenocarcinoma; CYFRA 21-1: cytokeratin 19 fragments; CA 19-9: carbohydrate antigen 19-9; + positive; −: negative; MST: median survival time

We included 246 patients with ALAD in the study (Fig. 2). Patient characteristics and comparison of clinical profiles of CA 19-9^+^ and CA 19-9^−^ patients are shown in Table 1. Their median age was 67 years (interquartile range, 61–75 years); 184 (75 %) patients had Eastern Cooperative Oncology Group Performance Status (ECOG PS) of 0 or 1; and 26 (11 %) patients had stage IIIB disease. Of the 246 patients, 100 (41 %) had *EGFR* mutations in their specimens, and 22 (9 %) had chronic lung inflammatory diseases (16 with interstitial pneumonia, 3 with NTM infection, and 3 with bronchiectasis). We found 163 (66 %) were CEA^+^ (>5.0 ng/ml), 155 (63 %) were CYFRA 21-1^+^ (>2.2 ng/ml) and 76 (31 %) were CA 19-9^+^ (>37.0 U/mL). Chemotherapy regimens of patients who did not receive platinum doublet therapy were tyrosine kinase inhibitors: *n* = 34; pemetrexed: *n* = 18; TS-1: *n* = 6; paclitaxel: *n* = 5; vinorelbine: *n* = 4; gemcitabine: *n* = 4; docetaxel: *n* = 3; and gemcitabine/vinorelbine therapy: *n* = 2.

**Table 1:** Characteristics and differences by serum CA 19-9 levels in patients with advanced-stage lung adenocarcinoma

Patient characteristics	Total *n* (%) (*n* = 246)	CA 19-9 positive *n* (%) (*n* = 76)	CA 19-9 negative *n* (%) (*n* = 170)	*P*
Age (years)				
SD	10.4	10.7	10.2	0.160
Mean	67.1	68.5	66.5	
Sex				
Male	154 (63)	47 (62)	107 (63)	0.887
Female	92 (37)	29 (38)	63 (37)	
Smoking status				
Never	101 (41)	29 (38)	72 (42)	0.577
Current or former	145 (59)	47 (62)	98 (58)	
ECOG PS				
0 or 1	184 (75)	46 (61)	138 (81)	<0.001
2–4	62 (25)	30 (39)	32 (19)	
Stage				
IIIB	26 (11)	6 (8)	20 (12)	0.501
IV	220 (89)	70 (92)	150 (88)	
*EGFR* status				
Mutated	100 (41)	30 (39)	70 (41)	0.888*
Exon 19 deletion	45 (18)	16 (21)	29 (17)	
Exon 21 point mutation	48 (20)	11 (14)	37 (22)	
Others	7 (3)	3 (4)	4 (2)	
WT or uninvestigated	146 (59)	46 (61)	100 (59)	
Inflammatory lung disease				
Present	22 (9)	10 (13)	12 (7)	0.147
Absent	224 (91)	66 (87)	158 (93)	
Serum CEA				
Positive	163 (66)	53 (70)	110 (65)	0.469
Negative	83 (34)	23 (30)	60 (35)	
Serum CYFRA 21-1				
Positive	155 (63)	59 (78)	96 (56)	0.002
Negative	91 (37)	17 (22)	74 (44)	
Chemotherapy				
Platinum doublet	170 (69)	43 (57)	127 (75)	0.007
Others	76 (31)	33 (43)	43 (25)	

*CA 19-9* carbohydrate antigen 19-9, *CEA* carcinoembryonic antigen, *CYFRA 21-1* cytokeratin 19 fragments, *ECOG PS* Eastern Cooperative Oncology Group Performance Status, *EGFR* epidermal growth factor receptor gene, *SD* standard deviation, *WT* wild-type

*Comparison between patients with mutated *EGFR* and those with WT or uninvestigated *EGFR*

Comparison of clinical profiles of CA 19-9^−^ and CA 19-9^+^ patients showed the CA 19-9^−^ group included a significantly higher percentage of patients with ECOG PS 0 or 1 status (*P* < 0.001), serum CYFRA 21-1^−^ (*P* = 0.002), and platinum doublet therapy administration (*P* = 0.007). *EGFR* status (*P* = 0.888) and presence of inflammatory lung disease (*P* = 0.147) did not statistically differ between the two groups.

Clinical profiles of CYFRA 21-1^−^ and CYFRA 21-1^+^ patients are compared in (Additional file 1: Table S1). The CYFRA 21-1^−^ group had a significantly higher percentage of patients with ECOG PS 0 or 1 status (*P* = 0.002), inflammatory lung disease (*P* = 0.020), serum CA 19-9^−^ (*P* = 0.002), and platinum doublet therapy administration (*P* = 0.046). *EGFR* status (*P* = 0.502) did not statistically differ between the two groups.

Test of correlation between CA 19-9 levels and CYFRA 21-1 levels showed no significant relationship between these tumor markers (*r* = 0.006). The scatter plot is shown in (Additional file 2: Figure S1).

### Survival analysis according to the tumor markers

**Fig. 3:**
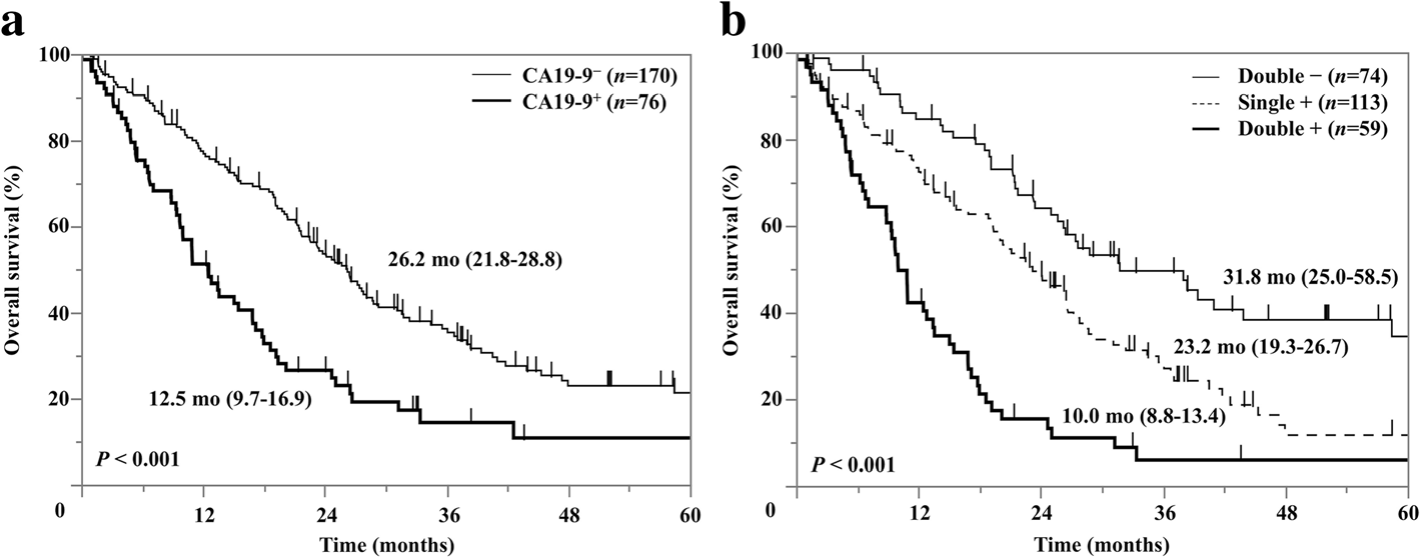
Kaplan–Meier curves for overall survival in patients with advanced stage disease, by (**a**) serum CA 19-9 positivity; and (**b**) both serum CA 19-9 and serum CYFRA 21-1 (Double −: neither marker; Single +: one marker; Double +: both markers)

The OS of patients included in this study was 21.4 months (interquartile range, 18.9 − 25.0 months; Table 2). Overall survival curves of CA 19-9^+^ and CA 19-9^−^ patients are shown in Fig. 3a. The OS of serum CA 19-9^+^ patients (*n* = 170, 69 %) was 12.5 months (95 % CI [CI]: 9.7 − 16.9 months); that of serum CA 19-9^−^ patients (*n* = 76, 31 %) was 26.2 months (CI: 21.8 − 28.8 months; *P* < 0.001). The OS of serum CYFRA 21-1^+^ patients (*n* = 155, 63 %) was 16.9 months (CI: 12.6 − 19.7 months), and that of serum CYFRA 21-1^−^ patients (*n* = 91, 37 %) was 31.8 months (CI: 26.2 − 43.9 months; *P* < 0.001).

**Table 2:** Analysis of overall survival time by clinical factors

Characteristics	Patients *n* (%)	OS (months)	Univariate analysis	Multivariate analysis	
			*P*	HR (95 % CI)	*P*
Age (years)					
≥ 75	62 (25)	15.1	0.005	1.35 (0.90–1.99)	0.150
< 75	184 (75)	24.2		Reference	
Sex					
Male	154 (63)	19.1	0.279	1.30 (0.87–1.97)	0.204
Female	92 (37)	26.2		Reference	
Smoking status					
Never	101 (41)	23.8	0.742	0.91 (0.64–1.36)	0.636
Current or former	145 (59)	19.3		Reference	
ECOG PS					
0 or 1	184 (75)	26.5	<0.001	0.42 (0.29–0.62)	<0.001
2–4	62 (25)	8.8		Reference	
*EGFR* status					
Mutated	100 (41)	31.1	0.001	0.41 (0.29–0.57)	<0.001
WT or uninvestigated	146 (59)	16.9		Reference	
Stage					
IIIB	26 (11)	43.9	0.016	0.38 (0.21–0.64)	<0.001
IV	220 (89)	20.2		Reference	
Serum CEA					
Negative	83 (34)	20.4	0.985	0.79 (0.56–1.11)	0.180
Positive	163 (66)	21.8		Reference	
Serum CYFRA 21-1					
Negative	91 (37)	31.8	<0.001	0.47 (0.32–0.66)	<0.001
Positive	155 (63)	16.9		Reference	
Serum CA 19-9					
Negative	170 (69)	26.2	<0.001	0.60 (0.43–0.85)	0.005
Positive	76 (31)	12.5		Reference	
Chemotherapy					
Platinum doublet	170 (69)	25.0	<0.001	0.64 (0.42–0.97)	0.034
Others	76 (31)	14.2		Reference	

*CA 19-9* carbohydrate antigen 19-9, *CEA* carcinoembryonic antigen, *CI* confidence interval, *CYFRA 21-1* cytokeratin 19 fragments, *ECOG PS* Eastern Cooperative Oncology Group Performance Status, *EGFR* epidermal growth factor receptor gene, *WT* wild-type, *HR* hazard ratio, *OS* overall survival

### Multivariate analysis of overall survival time

Cox’s multivariate regression analysis of the influence of clinical characteristics on survival outcomes indicated that ECOG PS 0 or 1 (HR: 0.42, CI: 0.29 − 0.62, *P* < 0.001), mutated *EGFR* status (HR: 0.41, CI: 0.29 − 0.57, *P* < 0.001), stage IIIB (HR: 0.38, CI: 0.21 − 0.64, *P* < 0.001), serum CYFRA 21-1^−^ (HR: 0.47, CI: 0.32 − 0.66, *P* < 0.001), serum CA 19-9^−^ (HR: 0.60, CI: 0.43 − 0.85, *P* = 0.005), and administration of platinum doublet therapy (HR: 0.64, CI: 0.42 − 0.97, *P* = 0.034) independently predicted longer OS. To clarify the relationships of chemotherapy regimens to OS, we analyzed the platinum doublet subcohorts with regard to OS; we found that CA 19-9^−^ independently predicted longer OS (HR: 0.54, CI:0.35 − 0.84, *P* = 0.007).

### OS by combined serum levels of CA 19-9 and CYFRA 21-1

We initially divided the patients into 4 groups by serum CA 19-9 and CYFRA 21-1 level: (a) both CA 19-9^+^ and CYFRA 21-1^+^ (59 patients, 24 %; median survival time [MST]: 10.0 months [CI: 8.8 − 13.4 months]), (b) CA 19-9^+^ and CYFRA 21-1^−^ (17 patients, 7 %; MST: 26.7 months [CI: 12.5 − * months]), (c) CA 19-9^−^ and CYFRA 21-1^+^ (96 patients, 39 %; MST: 22.5 months [CI: 16.9 − 26.6 months]), and (d) both CA 19-9^−^ and CYFRA 21-1^−^ (74 patients, 30 %; MST: 31.8 months [CI: 25.0 − 58.5 months]). Although the MSTs of (a) and (b) (*P* = 0.001) and that of (c) and (d) (*P* = 0.003) differed statistically, the difference between the two single-positive groups, (b) and (c), was not significant (*P* = 0.14). Therefore, we combined groups (b) and (c) into one single positive group. We also defined group (a) as double positive and group (d) as double negative (Fig. 2). Survival curves for these 3 groups are shown in Fig. 3b. Their MSTs were double positive: 10.0 months (CI: 8.8 − 13.4 months); single positive: 23.2 months (CI: 19.3 − 26.7 months); and double negative: 31.8 months (CI: 25.0 − 58.5 months; *P* < 0.001).

### Pathological findings and recurrence-free survival analysis

**Fig. 4:**
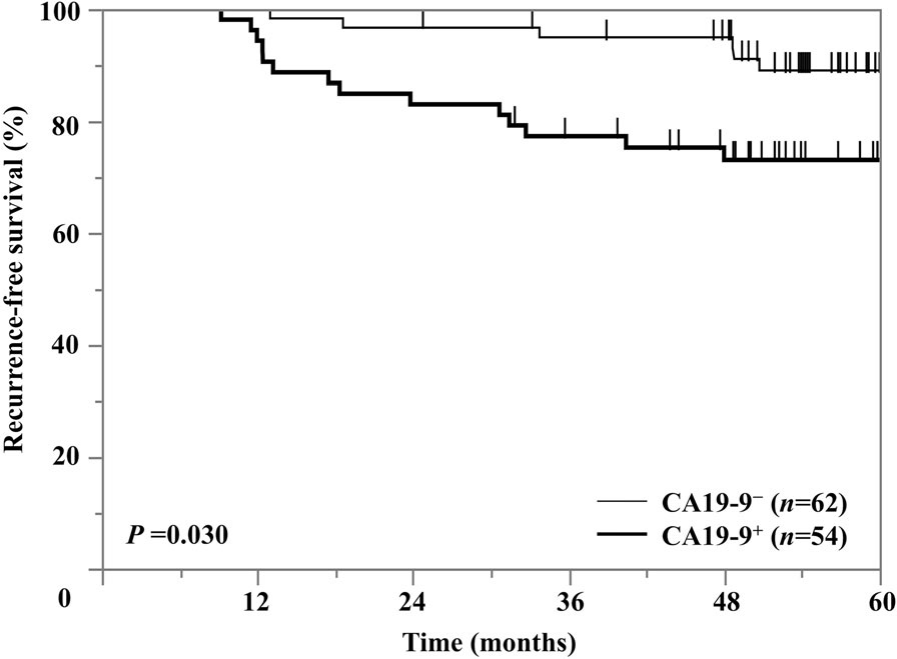
Kaplan–Meier curves for recurrence-free survival by histological CA 19-9 positivity in patients with stage I lung adenocarcinoma

We pathologically evaluated 116 consecutive surgically resected specimens from patients with clinical stage I lung adenocarcinoma. Of those, 54 (47 %) were CA 19-9^+^. Relationships between CA 19-9 expression and patients’ clinicopathological characteristics are summarized in Table 3. Comparison of clinical profiles of CA 19-9^+^ and CA 19-9^−^ patients showed the CA 19-9^+^ group to include significantly higher proportions of patients with vessel invasion (*P* = 0.032), pleural invasion (*P* = 0.023), cancer invasive factors (*P* = 0.005) and positive PAS stain (*P* = 0.001). CA 19-9^+^ patients had significantly shorter recurrence-free survival than CA 19-9^−^ patients (*P* = 0.030). The Kaplan-Meier curve is shown in Fig. 4. We also investigated the association between *EGFR* status and survival in the 116 patients with stage I lung adenocarcinoma; for whom 110 (95 %) had *EGFR* mutation analyses available, which showed 61 (53 %) to have *EGFR* mutations. Log-lank analysis revealed that *EGFR* status had no prognostic effect on recurrence-free survival (*P* = 0.569), or OS (*P* = 0.171).

**Table 3:** Relationships between serum CA 19-9 and clinicopathological factors in clinical stage I lung adenocarcinoma patients

Patient characteristics	CA 19-9 positive *n* (%) (*n* = 54)	CA 19-9 negative *n* (%) (*n* = 62)	*P*
Age (years)			
SD	9.1	8.3	0.028
Mean	69.2	65.6	
Sex			
Male	29 (54)	30 (48)	0.582
Female	25 (46)	32 (52)	
Smoking status			
Never	30 (56)	32 (52)	0.712
Current or former	24 (44)	30 (48)	
Vessel invasion			
Absent	39 (72)	55 (89)	0.032
Present	15 (28)	7 (11)	
Pleural invasion			
Absent	41 (76)	57 (92)	0.023
Present	13 (24)	5 (8)	
p-N status			
No metastasis	47 (87)	59 (95)	0.184
Metastasis	7 (13)	3 (5)	
Cancer invasive factor			
Negative	29 (54)	49 (79)	0.005
Positive	25 (46)	13 (21)	
c-Stage^a^			
IA	44 (81)	50 (81)	1.000
IB	10 (19)	12 (19)	
p-Stage^b^			
I	45 (83)	59 (95)	0.063
II/III	9 (17)	3 (5)	
PAS stain			
Negative	32 (59)	54 (87)	0.001
Positive	22 (41)	8 (13)	

*CA 19-9* carbohydrate antigen 19-9, *PAS* periodic acid-Schiff stain, *SD* standard deviation

^a^ Clinical stage; ^b^ Pathological stage

## Discussion

In the present study, we showed that both serum CA 19-9 and CYFRA 21-1 were independent prognostic markers in ALAD patients, and their combined use improves prognostic accuracy.

We have shown serum CA 19-9 to be an independent predictive factor for OS, according to multivariate analysis of possible prognostic factors that included serum CYFRA 21-1. To the best of our knowledge, this is the first report to show the correlation between positive CA 19-9 levels and shorter OS in patients with ALAD, although this correlation has been reported in adenocarcinomas of other organs such as pancreas, colon, and stomach [8–15]. The consistency of this pattern among adenocarcinomas of different organs implies that serum CA 19-9 might be a prognostic marker in all kinds of adenocarcinoma.

The major advantage of CA 19-9 is that it can be measured quickly at low cost. Additionally, CA 19-9 is a standard biomarker for gastrointestinal cancers, such as pancreatic, colon, and gastric cancers. Therefore we speculate that its application to lung cancer would be relatively easy.

Our results also showed that 31 % of ALAD patients had positive serum CA 19-9. We believe that this positive rate is common in patients with ALAD, although it was much lower than in studies of patients with advanced pancreatic adenocarcinoma (for which CA 19-9 is a prognostic marker), who were reportedly 50 % − 84 % positive [8, 10, 30].

The combined use of CA 19-9 and CYFRA 21-1 offers more accurate prognoses in patients with lung adenocarcinoma. In our study, as patients who were either CA 19-9^+^ or CYFRA 21-1^+^ did not significantly differ in survival, we regarded them as one group (single positive patients). Consequently, we divided patients into three groups as well; 24 % double positive (CA 19-9^+^/CYFRA 21-1^+^), 46 % single positive (either CA 19-9^+^ or CYFRA 21-1^+^), and 30 % double negative (CA 19-9^−^/CYFRA 21-1^−^). Survival curves for these 3 groups revealed significant relationships between these tumor markers and prognosis (Fig. 3b).

The precise reason for high CA 19-9 levels is unclear. However, large studies have shown that healthy volunteers did not have high serum CA 19-9 levels [31, 32]. High CA 19-9 elevation has been reported in some chronic inflammatory lung diseases, such as intestinal pneumonia, NTM infection, bronchiectasis, and diffuse panbronchiolitis [33]. In the present study, CA 19-9 positivity and presence of inflammatory disease showed no correlation (*P* = 0.147); thus inflammatory disease did not cause CA 19-9 elevation. In addition, our pathological analysis demonstrated that the lung cancer cells generated CA 19-9. Therefore, we speculated the elevated serum CA 19-9 was associated with the CA 19-9 generated by cancer cells.

Although CA 19-9 expression is also related to unfavorable prognosis in some kinds of cancer, why high CA 19-9 predict shorter OS is not understood [34, 35]. We therefore investigated the relationship between CA 19-9 IHC positivity and pathological findings in patients with stage I lung adenocarcinoma. We analyzed the presence of cancer invasive factors (vessel and pleural invasion) and mucin production, which were reportedly associated with highly malignant features such as shorter recurrence-free survival and OS in lung adenocarcinoma patients [24, 29, 36]. In our pathological analysis of clinical stage I patients, CA 19-9 positive lung adenocarcinoma had more histologically malignant features (*P* = 0.005) and shorter recurrence-free survival (*P* = 0.030) than CA 19-9 negative lung adenocarcinoma (Fig. 4). These findings indicate that CA 19-9 positive lung adenocarcinoma is highly malignant. We speculate that these malignant features caused the elevated serum CA 19-9, as cancer cell invasion to the blood could cause the elevated serum tumor markers.

Our findings are of special interest, but there were some limitations to this study. First, as this study was conducted in a single institute, it included limited number of patients. Second, a considerable number of patients were excluded from this analysis due to missing tumor marker data before their initial therapy, because measurement of tumor markers was at the discretion of the attending physician. Third, subject selection in this study was confined to Japanese patients, and racial differences may need to be considered in the interpretation of this study. Fourth, we did not examine Lewis antigen status [37]. Patients who are Lewis antigen-negative cannot synthesize CA 19-9, and therefore present as falsely negative [38]. However, as they comprise only 5–7 % of the general population, we assume that it did not affect the results [39].

## Conclusions

In conclusion, our study showed serum CA 19-9 to be an independent prognostic indicator in patients with ALAD, and combined use of CA 19-9 and CYFRA 21-1 to provide more accurate prognostic information.

## Additional files

Additional file 1: Table S1. Characteristics and differences by serum CYFRA 21-1 levels in patients with advanced-stage lung adenocarcinoma. (DOCX 24 kb)
Additional file 2: Figure S1. Scatter-plot of serum CA 19-9 and CYFRA 21-1 levels. (JPG 394 kb)

## Abbreviations

ALAD: Advanced lung adenocarcinoma
CA 19-9: Carbohydrate antigen 19-9
CEA: Carcinoembryonic antigen
CI: Confidence interval
CLEIA: Chemiluminescent enzyme immunoassay
CYFRA 21-1: Cytokeratin 19 fragments
ECOG PS: Eastern Cooperative Oncology Group Performance Status
*EGFR*: Epidermal growth factor receptor gene
HR: Hazard ratio
IHC: Immunohistochemical
MST: Median survival time
NSE: Neuron-specific enolase
NTM: Non-tuberculous mycobacteriosis
OS: Overall survival
PAS: Periodic acid Schiff
SD: Standard deviation
TNM: Tumor, node, metastasis
ULN: Upper limit of normal
