# Supplementary material for: The prognostic value of serum CA 19-9 for patients with advanced lung adenocarcinoma
Source: BMC Cancer. 2016 Nov 14;16:890. doi: 10.1186/s12885-016-2897-6 (PMC5109711; doi:10.1186/s12885-016-2897-6)
Supplement: Additional file 1: Table S1. — Characteristics and differences by serum CYFRA 21-1 levels in patients with advanced-stage lung adenocarcinoma. (DOCX 24 kb) [file 12885_2016_2897_MOESM1_ESM.docx]

**Supplementary Table 1**. Characteristics and differences by serum CYFRA 21-1 levels in patients with advanced-stage lung adenocarcinoma

| **Patient characteristics** | **Total**  ***n* (%)**  **(*n*=246)** | **CYFRA 21-1 positive**  ***n* (%)**  **(*n*=155)** | **CYFRA 21-1 negative**  ***n* (%)**  **(*n*=91)** | ***P*** |
| --- | --- | --- | --- | --- |
| **Age (years)** |  |  |  |  |
| SD | 10.4 | 10.6 | 9.8 | 0.125 |
| Mean | 67.1 | 67.9 | 65.8 |  |
| **Sex** |  |  |  |  |
| Male | 154 (63) | 103 (66) | 51 (56) | 0.133 |
| Female | 92 (37) | 52 (34) | 40 (44) |  |
| **Smoking status** |  |  |  |  |
| Never | 101 (41) | 57 (37) | 44 (48) | 0.082 |
| Current or former | 145 (59) | 98 (63) | 47 (52) |  |
| **ECOG PS** |  |  |  |  |
| 0 or 1 | 184 (75) | 106 (68) | 78 (86) | 0.002 |
| 2–4 | 62 (25) | 49 (32) | 13 (14) |  |
| **Stage** |  |  |  |  |
| IIIB | 26 (11) | 15 (10) | 11 (12) | 0.668 |
| IV | 220 (89) | 140 (90) | 80 (88) |  |
| ***EGFR* status** |  |  |  |  |
| Mutated | 100 (41) | 66 (43) | 34 (37) | 0.502* |
| Exon 19 deletion | 45 (18) | 31 (20) | 14 (15) |  |
| Exon 21 point mutation | 48 (20) | 31 (20) | 17 (19) |  |
| Others | 7 (3) | 4 (3) | 3 (3) |  |
| WT or uninvestigated | 146 (59) | 89 (57) | 57 (63) |  |
| **Inflammatory lung disease** |  |  |  |  |
| Present | 22 (9) | 19 (12) | 3 (3) | 0.020 |
| Absent | 224 (91) | 136 (88) | 88 (97) |  |
| **Serum CEA** |  |  |  |  |
| Positive | 163 (66) | 107 (69) | 56 (62) | 0.264 |
| Negative | 83 (34) | 48 (31) | 35 (38) |  |
| **Serum CA 19-9** |  |  |  |  |
| Positive | 76 (31) | 59 (38) | 17 (19) | 0.002 |
| Negative | 170 (69) | 96 (62) | 74 (81) |  |
| **Chemotherapy** |  |  |  |  |
| Platinum doublet | 170 (69) | 100 (65) | 70 (77) | 0.046 |
| Others | 76 (31) | 55 (35) | 21 (23) |  |

CYFRA 21-1: cytokeratin 19 fragments; CEA: carcinoembryonic antigen; CA 19-9: carbohydrate antigen 19-9; ECOG PS: Eastern Cooperative Oncology Group Performance Status; *EGFR*: epidermal growth factor receptor gene; SD: standard deviation; WT: wild-type.

* Comparison between patients with mutated *EGFR* and those with WT or uninvestigated *EGFR*.
