# Supplementary figures and images for: The prognostic value of serum CA 19-9 for patients with advanced lung adenocarcinoma
Source: BMC Cancer. 2016 Nov 14;16:890. doi: 10.1186/s12885-016-2897-6 (PMC5109711; doi:10.1186/s12885-016-2897-6)

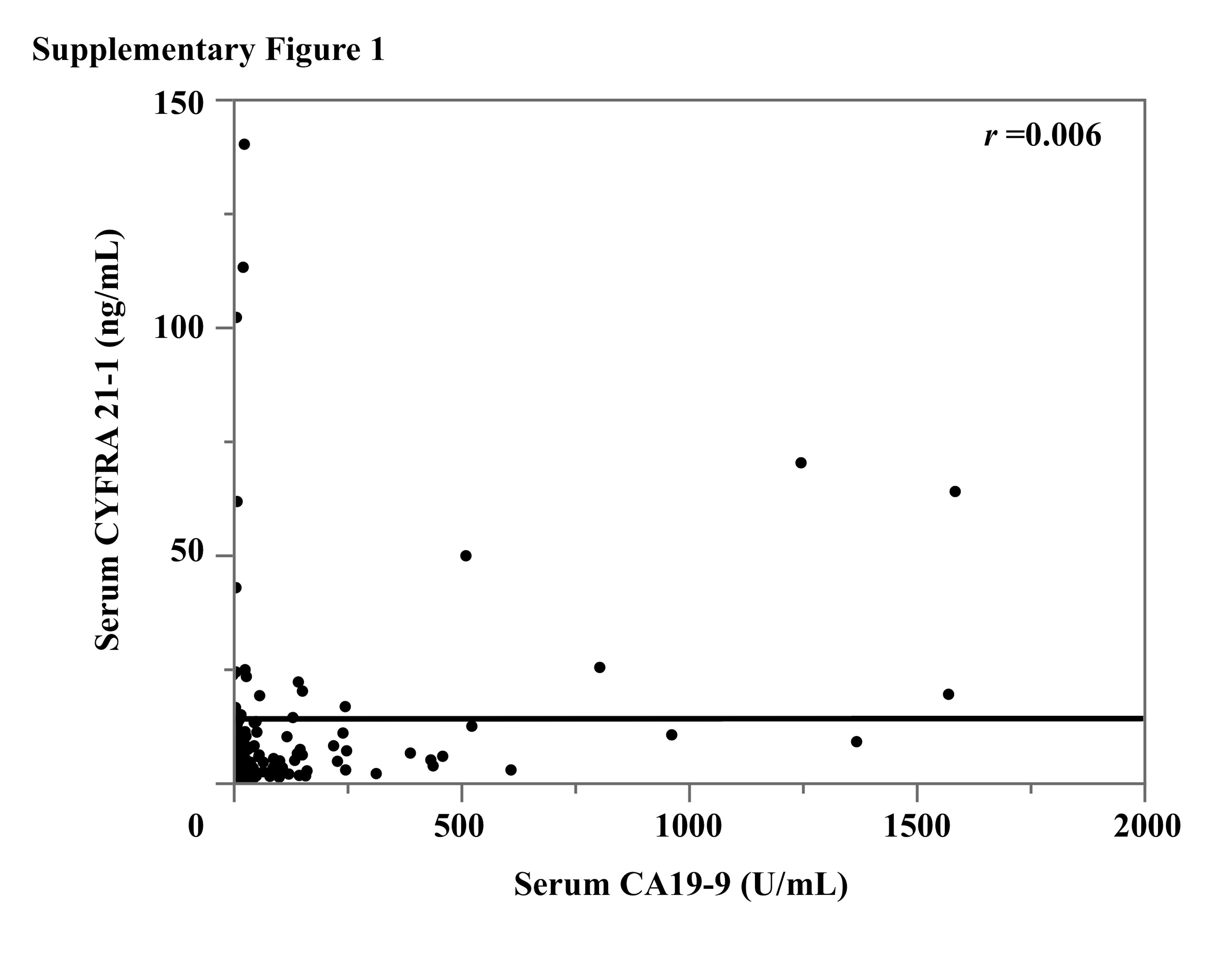

Supplement: Additional file 2: Figure S1. — Scatter-plot of serum CA 19-9 and CYFRA 21-1 levels. (JPG 394 kb) [file 12885_2016_2897_MOESM2_ESM.jpg]
